# Exploring the Posterolateral Corner of the Knee Joint: A Detailed Review of Recent Literature

**DOI:** 10.3390/jcm14051549

**Published:** 2025-02-25

**Authors:** Assala Abu-Mukh, Seungyup Lee, Hye Chang Rhim, Ki-Mo Jang

**Affiliations:** 1 Department of Orthopedics and Traumatology, IRCCS San Raffaele Hospital, Vita Salute San Raffaele University, 20132 Milan, Italy; assala.abumukh@gmail.com; 2 Department of Orthopedic Surgery, Anam Hospital, Korea University College of Medicine, Seoul 02841, Republic of Korea; yup101910@gmail.com; 3 Department of Physical Medicine and Rehabilitation, Harvard Medical School, Spaulding Rehabilitation Hospital, Boston, MA 02115, USA; hrhim@mgh.harvard.edu; 4 Department of Sports Medical Center, Anam Hospital, Korea University College of Medicine, Seoul 02841, Republic of Korea

**Keywords:** knee joint, posterolateral corner, fibular collateral ligament, popliteus tendon, popliteofibular ligament, posterolateral corner reconstruction

## Abstract

One of the least understood and most elaborate and neglected knee stabilizers is the posterolateral corner (PLC) complex. PLC injury is associated with a high risk of re-injury, early athletic career termination, instability, progressive osteoarthritis, and a high risk of artificial knee replacement. The growing focus on the PLC, along with various recent anatomical and biomechanical studies, has provided further insights into the anatomy and function of posterolateral structures in knee stabilization and kinematics. The PLC should be considered as a functional unit, not only an anatomical unit. A low suspicion threshold should be maintained when considering PLC injuries, and thorough history evaluation, clinical examination, and adequate imaging should be conducted to reduce the chances of neglected PLC injuries. Various PLC repair and reconstruction techniques, ranging from non-anatomical to anatomical, have been introduced, with treatments increasingly favoring minimal incisions and arthroscopic procedures. Recent studies on the PCL have reported an increasing number of satisfactory clinical outcomes. This study aimed to provide a deeper understanding, as well as review the current and most feasible treatments for PLC injuries.

## 1. Introduction

Knee stability is the single most important factor affecting knee functionality and daily living and is a manifestation of the balance between numerous anatomical and functional structures [1,2,3]. Whenever knee balance is disrupted, consequent trauma and morphological adaptations, whether microscopic or macroscopic, occur [4].

One of the least understood and most elaborate stabilizers of the knee is the posterolateral knee corner (PLC) complex, which was first described by Hughston as a posterolateral stabilizer in an isolated posterior cruciate ligament resected specimen [5]. Since then, PLC has gradually become more of a focus, and various clinical tests and treatment methods for PLC injuries have been employed [6]. Nevertheless, the literature indicates that surgeons tend to focus on cruciate and collateral ligament injuries, whereas PLC injuries are often considered secondary, or neglected altogether [7].

The resulting consequences of PLC neglect are important; chronic posterolateral knee instability is probable, and the average delay in diagnosis is estimated to be approximately 30 months [8]. Neglected PLC injuries are associated with a high risk of re-injury, which is crucial, especially when considering athletic settings, as they may lead to early career termination. Instability leads to a reduction in the quality of life and consequent disability, progressive medial knee osteoarthritis (OA), biomechanical malalignment associated with varus thrust, and eventually, a high chance of undergoing total knee arthroplasty [9].

Typically, the mechanism of injury is described as a direct blow to the knee, either in an extended or a flexed position, with an associated varus-directed force. Nevertheless, other injury mechanisms including valgus-directed forces have been described to cause PLC injury.

Injuries of the PLC may vary in extent and severity, and their treatment should be consequently adjusted to the type of injury. Unfortunately, there is limited literature addressing the conservative management of PLC injuries; the conservative approach fails in higher-grade injuries, while it often leads to an acceptable clinical outcome in low-grade PLC injuries [10]. Since PLC injuries are often the outcome of sports injury or motor vehicle accidents, patients affected with PLC injuries tend to be young and present elevated functional demands, requiring dedicated care, a timely diagnosis, an adequate surgical choice, as well as appropriate postoperative management.

This review aims to gather and provide a deeper understanding, as well as review the current and most feasible treatments for PLC injuries reported over the past five decades [11,12,13].

## 2. Epidemiology

PLC injuries were considered uncommon [14]; however, recent research has shown that PLC injuries are highly underestimated owing to the difficulty of diagnosis and scarce attention [15].

The PLC complex is proven to be frequently injured in acute and chronic settings. In a magnetic resonance imaging (MRI) study, LaPrade et al. [15] reported that PLC was damaged in 16.04% of patients exhibiting acute ligament injuries and in 9.1% of all acute knee injuries presenting hemarthrosis. Acute PLC injuries are rarely present in an isolated form (2.1%), but most (86.67%) are present in the context of combined tears. The associated tears include the anterior cruciate ligament (ACL), medial collateral ligament (MCL), and posterior cruciate ligament (LCP) [15].

There is limited information regarding chronic PLC insufficiency; nevertheless, it is a result of both or either an acute or neglected morphology, such as a varus knee deformity, in which repetitive knee use leads to posterolateral structural laxity and long-term knee instability [16].

There is a subsequent predisposition to injuries and long-term OA in the context of knee instability, which constitutes a predictable economic burden worldwide [17]. Therefore, a systematic understanding and a timely management of PLC injuries are mandatory.

## 3. Anatomy and Biomechanics

The PLC of the knee joint confers stability to the posterolateral knee through the implication of different anatomical structures throughout the range of motion (ROM). The synergistic effect resulting from multiple anatomical structures forming the posterolateral knee is referred to as a functional unit.

The anatomical components of the PLC are divided into static stabilizers (a) and dynamic stabilizers (b), which guarantee primary knee stability through their passive anatomical structures. Dynamic stabilizers guarantee knee stability via their variable tension and anatomical relationships throughout the range of motion.

Static PLC stabilizers include the fibular collateral ligament (FCL), popliteus tendon (PT), popliteofibular ligament (PFL), arcuate complex (AC), and posterolateral capsule (PC). Dynamic PLC stabilizers include the iliotibial band (ITB), biceps femoris tendon (BFT), lateral gastrocnemius muscle (LGM), and popliteus muscle.

Each component contributes to stability at different degrees of knee flexion, maintaining stability throughout the entire range of motion. As an anatomical structure, the PLC stabilizes the knee against varus stress (1), tibial rotation (2), and tibial translation (3).

(1) Stability against varus stress is primarily provided by the FCL, while other structures contribute to it as a secondary function [18].
(2) Rotational stability is divided into external and internal tibial rotations. Stability against external rotation is provided mostly by the PFL and PT at higher degrees of flexion [19] and by the deep layer of the ITB, the PC, and the AC at lower knee flexion degrees, while resistance to internal rotation is guaranteed by the BFT and superficial layer of the ITB at higher flexion [20].
(3) Stabilizers against tibial translation are divided into anterior and posterior stabilizers, and posterior stability provided by the PLC reduces the strain on the PCL and is guaranteed mostly by the popliteus complex at approximately 90° [21] and by the AC [22,23]. PC seems to stabilize the knee at early degrees of flexion [24], while biceps femoris long-head activation offers resistance against anterior tibial translation before 40° [25], and both layers of the ITB restrain anterior tibial translation throughout the ROM [20].

In complex terms, the overall load supported by the functional unit was demonstrated to be slightly reduced at 120° compared with 90° of knee flexion [26] through a simulation-based model on a reconstructed PLC [27].

Our review mainly focuses on the three main structures: FCL (1), PT (2), and PFL (3).

(1) The FCL has been the focus of many biomechanical studies, and its main function is to resist varus stress at all knee flexion degrees [28]. Furthermore, it is a constantly considered area when reconstructing the PLC using the different techniques discussed in the Section 7 of this paper. In addition to varus stability, the FCL has a secondary function of rotational stability, which is more noticeable at early flexion angles (0–30°) [29]. The FCL exerts multidirectional stabilization over the tibial translation during the stance, and its effects have been thoroughly discussed by Smith et al. [30].

Regardless of their function, there seems to be a complementary shifting tendency in which most posterolateral knee structures exhibit around 90° of flexion; structures that are initially taught at early stages become minimally influential beyond 90° and vice versa; and structures that are lax become maximally taught around 90° [29,31,32].

(2) The PT (which generates from the popliteus muscle) was defined as “the fifth ligament of the knee” and is considered as both a static and dynamic stabilizer. The PT has the primary function of stabilizing the PLC, especially against external rotation, which is mostly exploited above 90° [19]. PT also plays a secondary role in limiting posterior tibial translation throughout knee flexion. Even though PT function is prominent above 90°, it plays a crucial role in full extension as it is responsible for unlocking the gait through its muscular contraction to internally rotate the tibia and externally rotate the femur with respect to one another [33].
(3) The PFL primarily stabilizes the functional unit against external tibial rotation, varus angulation, and anterior tibial translation through its synergetic function in conjunction with the FCL and PT. Also, PFL sectioning leads to an increase in the ACL load at all degrees of knee flexion [34,35,36,37].

The anatomical origins, insertions, and roles of the functional unit components are summarized in Table 1.

## 4. Clinical Examination

Knee examination is elaborate and often directed towards a pre-existing clinical suspicion. The most crucial part of any orthopedic examination is clinical history; from this point, a hypothesis of acute versus chronic pathologies can be drawn. When addressing PLC injuries, both alignment and anamnesis should be assessed accurately. A history of sports injury or motor vehicle accidents are often reported; those include a varus-directed knee blow or externally rotated distortion or a direct blow to the anteromedial tibia. A clinical suspicion of PLC injury can be formulated based on the time and specific mechanism of the traumatic event. In acute cases, the majority of patients exhibit tenderness in the posterolateral knee aspect [51]. PLC injuries are often multiligamentous and may require a more complex clinical evaluation as well as more elaborate diagnostic examinations. In acute settings, instabilities should be assessed using multiple clinical tests and not solely through a single test, because sensitivity, specificity, and operator dependency all influence the reliability of clinical tests. The use of multiple tests correlates with a more valid clinical suspicion; however, in acute settings, test conduction is limited because of pain and apprehension.

Whenever no trauma history is present, the careful assessment of the lower limb alignment should be considered. In fact, varus limb alignment strongly correlates with structural PLC insufficiency and indicates chronic mechanical joint abuse, which often requires a multistep approach.

### 4.1. Gait

A physical examination begins with gait examination; if the patient’s limb alignment is varus, chronic insufficiency is likely. Gait assessments should consider the presence of varus thrust and knee hyperextension, which usually indicate higher mechanical loads in the medial compartment throughout the gait cycle, especially in younger patients [52]. 

### 4.2. Evaluating Instability

Anatomical structures work in a synergetic balance that results in functional stability. Therefore, evaluating instability patterns is of pivotal importance for understanding which anatomical structures may be severed. Clinically, instability is often reported as a sensation of the knee “giving way”; thus, determining what structure contributes primarily to the patient’s symptoms can be challenging, as isolated structural injuries may individually contribute less to the overall clinical picture [53].

### 4.3. Tests

Varus: laxity at 0° might indicate a combined injury of one cruciate ligament with the FCL, whereas laxity at 30° indicates an isolated FCL injury [54,55].

Dial test: performed in the prone position and when there is external rotation limb asymmetry over 10° on a 30° flexed knee, it indicates PLC injury; when the test is positive at 90° of knee flexion, it indicates an associated PCL injury [54,56].

The reverse pivot test is conducted with the knee flexed at 90°, with external tibial rotation and a valgus-directed force. Throughout knee extension, the ITB reduces the tibia to its anatomic anterior position with a palpable or audible clunk owing to the ITB shift from a flexor to extensor around 20–30°. This test has a high false-positive rate [57].

Posterolateral drawer test: This is a reliable test performed at 45° of hip flexion, 80° of knee flexion, and 15° of foot external rotation. A laterally rotated and posteriorly directed force is applied over the proximal tibia and compared with the contralateral side [55].

External rotation recurvatum test: By holding the foot from the toes while the patient is in a supine position, if the PLC is injured, the proximal tibial falls in an externally rotated position. This sign is frequently present in combined cruciate injuries; however, it has low sensitivity [55].

Evaluating ankle dorsiflexion and sensation is crucial, and their alterations indicate peroneal nerve injury, which affects as many as 31% of patients [58].

The recent literature provides various classification systems for PLC injuries based on the severed structures, the extinct of injury, and its timing, simplifying the identification of an adequate treatment algorithm [59]. Weiss et al. [60] classify PLC injuries into four types based on the involved structures and their consequent instabilities: Type 1 injury involves PCL rupture and consequent posterior instability; Type 2 involves an additional tear of the PFL, leading to minor postero-lateral rotatory instability; Type 3 requires an additional partial FCL injury causing posterior, rotatory, and lateral-directed instabilities; and Type 4 requires a total FCL tear and possible additional structure involvement leading to associated posterior, rotatory, and major lateral instabilities.

When considering cases with intact PCL, the severity of PLC instability may be graded as I, II, or III if the lateral joint opening (or external rotation) is 0–5 mm (0–5 degrees), 6–10 mm (6–10 degrees), or above 10 mm (10 degrees), respectively, when compared with the contralateral knee [5]. Following a recently published treatment algorithm, Grade I injuries and grade II isolated injuries may be treated conservatively, estimating that high-demand patients with grade II injuries may undergo primary surgical repair. Acute and combined grade II injuries (below 3 weeks) may benefit from repair, while grade II and grade III injuries should be assessed for chronicity and the presence of associated malalignment which often indicates eventual staged osteotomy and PLC component reconstruction [59].

## 5. Diagnostics

Once a clinical hypothesis has been formulated, relative imaging should be performed to confirm and accurately plan a treatment algorithm for each patient. Less often, imaging may be the first indicator of pathological posterolateral corner behavior.

### 5.1. Plain Radiographs

Radiographs are accessible, provide good information regarding lower limb alignment, and are crucial when planning a high tibial osteotomy (HTO) for chronic malalignment associated with posterolateral insufficiency. A semi-dynamic study can be conducted through stress radiographs and provide additional information regarding lateral joint line opening; whenever the difference between the medial and lateral joint lines is > 4 mm, PLC involvement can be diagnosed [61]. Associated cruciate ligament insufficiency can be evaluated by using anterior and posterior stress radiographs.

### 5.2. Magnetic Resonance Imaging (MRI)

MRI is indispensable for ligamentous status evaluation as it can indicate which structures are injured and provides additional information regarding cruciate and collateral ligaments, bone bruising, and meniscal involvement, which generally indicate a severe traumatic event and a deteriorated biological environment [62,63]. Transverse, sagittal, and coronal scans are helpful in evaluating the FCL, which can be identified as a low-signal structure connecting the lateral femoral epicondyle with the fibular head; in some cases, it merges with the biceps femoris tendon to form a conjoint structure [64].

PLC injuries should be suspected in the presence of peri-ligamentous edema, intrasubstance ligament tears, and fibular head avulsion or fractures [65,66,67].

In cases of PT or muscle involvement, avulsions may rarely occur; however, the involvement of the muscle belly or musculotendinous junction is more common [68]. The PT is visualized as a low-to-intermediate-signal structure on transverse or coronal images [69].

PFL is consistently present; however, its visualization is possible through the use of alternative scans. The PFL appears as a low-intensity structure on coronal or sagittal imaging. The PFL identification rate is approximately 8%; however, when coronal oblique planes are used, it is identified in 53% of cases [70]. Injury to PLC structures may be seen as an increased signal within the ligament, discontinuity, or avulsion [69].

### 5.3. Computerized Tomography

Computerized tomography (CT) is not a primary test used in the diagnosis of PLC injuries; however, CT is more accessible and rapidly conducted compared to MRI and provides a different set of information compared to other studies, which is beneficial when considering avulsion fractures, tunnel sizing [71], and associated pathologies such as trochlear dysplasia [72]. When planning surgery, ultra-low-dose CT scans have superior accuracy for detecting non-displaced fractures and performing bone measurements [73].

### 5.4. Arthroscopic Diagnosis

A definitive diagnosis of joint pathologies generally requires direct arthroscopic visualization and probing. Arthroscopy is a validated treatment method for PLC injuries, and arthroscopic maneuvers can confirm the involvement of the PLC through the lateral gutter and posterolateral drive-through signs, which are indirect indicators of PLC injury [74]. The three pillars of the PLC present arthroscopically identifiable landmarks, and the acute signs of injury may be directly visualized during arthroscopy [6].

## 6. Conservative Management

From the limited number of studies addressing the conservative management of PLC injuries, it emerges that conservative management may be beneficial and feasible for lower-grade PLC injuries, while it leads to a poor quality of life and clinical outcome in higher-grade injuries [10]. In the later study, instability is defined as a lateral opening of the affected joint compared with the contralateral knee during stress maneuvers. Instability is graded as I if the lateral joint opening is 0–5 mm, grade II if a 6–10 mm opening is present, and grade III if the opening is above 10 mm compared with the contralateral side.

Conservative management (immobilization for 2–5 weeks) in grade II instability led to full recovery in 82% of cases with an average 88 Lysholm score, while the conservative approach (immobilization for 2–7 weeks) in grade III instability led to a 75% decreased activity level with a mean of a 65 Lysholm score [75].

Considering that most PLC injuries occur in sports events and in young patients, whether opting for conservative or surgical management, it is crucial to address the consequent return to sports and previous activity levels through the evaluation of muscle performance and a dedicated rehabilitation protocol [76].

The rehabilitation should consider acquiring progressive QM control and ROM, establishing normal gait patterns, and initiating a plyometric training program that should be modulated based on involved structures and injury timing [77].

## 7. Surgical Techniques

Various reconstruction techniques have been described in the literature. Posterolateral knee corner reconstruction does not guarantee the restoration of the functional unit; however, it is mandatory to regain the functional aspect of the posterolateral knee corner.

Surgical techniques are divided into three categories: (1) non-anatomic treatment, (2) anatomic reconstruction, and (3) arthroscopic reconstruction. The most important surgical reconstructions are summarized in Table 3.

(1) Non-anatomical Treatment (NAT)

NAT consists of treatments, repairs, and reconstructions that are often performed through open surgery, and its goal is to provide partial functional stability rather than anatomical stability. NATs include biceps tenodesis and rerouting [78,79], an extra-articular ITB sling [80], arcuate complex and bone block advancements [14,81,82], as well as reconstruction through a variable number of tibial or femoral tunnels [83].

- BFT rerouting to achieve posterolateral stability was first described in 1988 [84]. It started as tenodesis by repositioning the BFT insertion 1 cm anterior to the FCL origin at the femur and was effective in restoring the knee varus and external rotation stability. However, in the same study, overconstraints of the varus and external rotation were reported [78]. NAT then evolved into biceps rerouting, which was used until recently [85,86].
- NAT techniques include ligament avulsion repairs through advancement into a bony bed, and intrasubstance tear repair through sutures made in a “pants over vest” fashion [51]. The combined advancement of the lateral gastrocnemius, posterolateral capsule, and arcuate complex was performed and reported by Hughston in 1985 using a single staple to connect and insert all three structures over the lateral femoral condyle [14].

**Figure 1:**
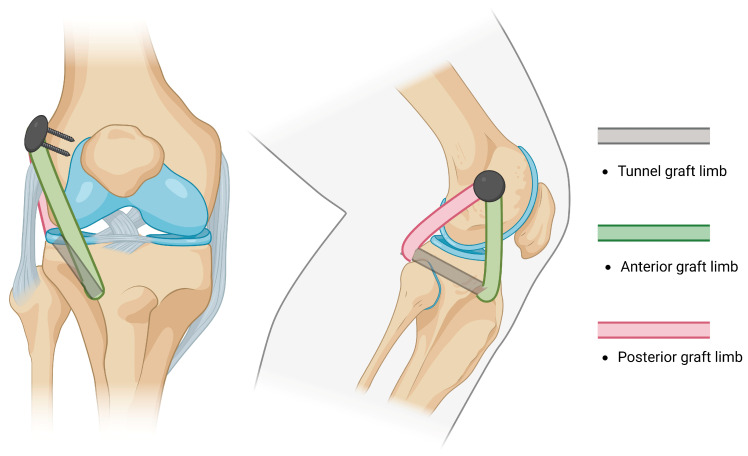
An illustration of the Albright technique: 6–8 mm tunnel is drilled adjacent to Gerdy’s tubercule. The posterior end of the tunnel is located 1–1.5 cm below joint surface and 1 cm medial to the proximal tibio-fibular joint.

**Figure 2:**
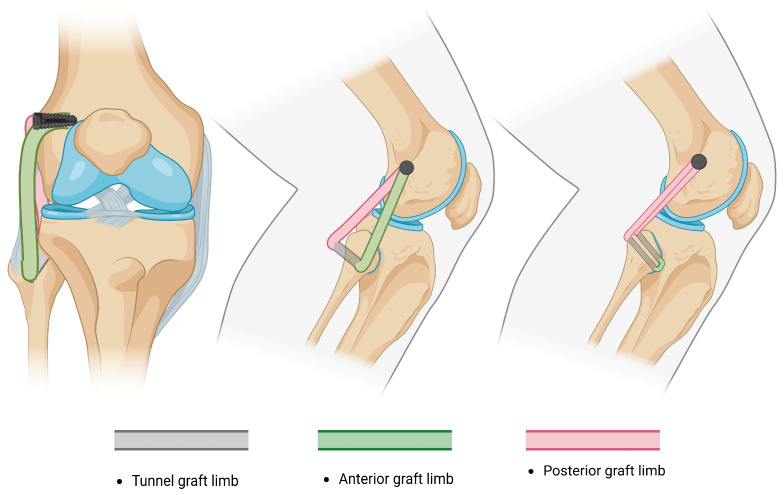
An illustration of the Fanelli–Larson technique: 6 mm tunnel is drilled through the fibular head in direction of the peroneal nerve. In cases of preserved varus and significant posterolateral instability, both limbs are passed posteriorly.

**Figure 3:**
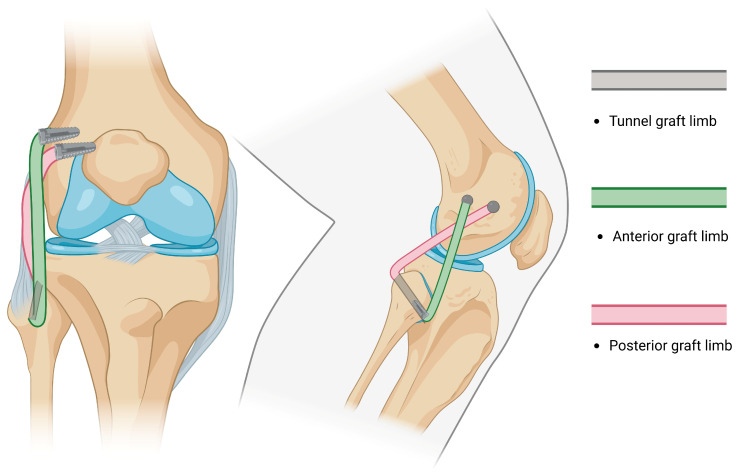
An illustration of the modified Larson technique: each graft end is fixed separately at the femoral insertion points to maintain isometry.

NATs evolved from repairs and advancements into widely used non-anatomical reconstructions [87,88] of the PLC by performing a single tibial tunnel and fixing both limbs of the graft in an isometric point at the femoral condyle described by J.P. Albright [83,89] (Figure 1). A reconstruction of the PLC through a single fibular tunnel and single femoral fixation site was afterwards described by Fanelli and Larson [90]; they report that in cases with preserved varus stability, both ends of the graft may be passed posteriorly, either by performing two parallel fibular tunnels or by fixing the doubled graft at the tibia and femur (Figure 2). Currently, the most likely reconstruction method of the PLC is a modification of the Fanelli–Larson technique [91,92,93,94] that tends towards the anatomy, in which each of the anterior and posterior graft limbs are passed from and fixed at the single fibular tunnel and inserted into two separate femoral sockets to guarantee differential graft limb tension (Figure 3).

The recent literature reports a variable yet higher failure rate of NATs than that of anatomical reconstructions [95]. We believe that this is because of the wide heterogeneity of NATs and the limited restoration of the functional unit. NAT overlooks knee complexity and bypasses the functional aspects that the knee requires throughout its ROM. Overall, NATs represent the “first generation surgeries” performed for PLC injuries, and mostly consist of techniques that are less used today as they do not restore the functional unit nor its anatomy, and provide inferior outcomes compared with anatomic reconstruction techniques, especially when considering high-demand, young patients.

(2) Anatomic reconstructions (AR)

The literature has shifted its focus towards AR, which aims to restore the anatomical structure of the FCL, PFT, and PT in various combinations, resulting in both anatomical and functional reconstructions. Nevertheless, AR also seems to provide partial results and is usually performed through open procedures. Commonly performed AR is addressed as anatomical; however, AR generally reconstructs only one or two components of the three aforementioned structures owing to surgical and anatomical limitations.

**Figure 4:**
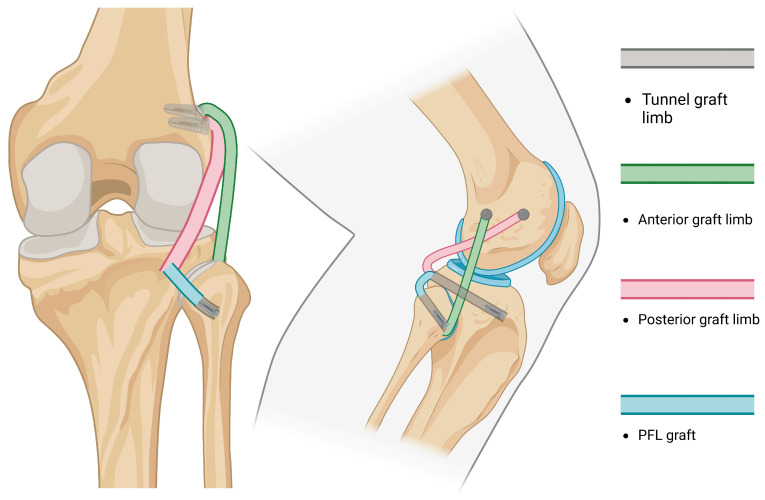
An illustration of LaPrade reconstruction: fibular and tibial tunnels are drilled, two femoral insertion sites are required for reconstruction, and popliteofibular ligament is reconstructed.

**Figure 5:**
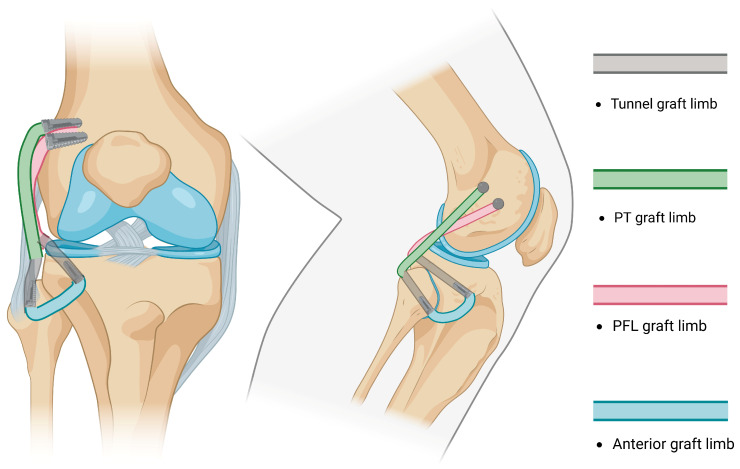
An illustration of Kim reconstruction (modified Hughston): Fibular and tibial tunnels are drilled and two femoral insertion sites are required for reconstruction. Both ends of the graft are passed from the posterior knee aspect.

- The Arciero technique reported the use of a single 7 mm trans-fibular tunnel, and the limbs of the graft are fixed into the femoral sockets through passage from the popliteal hiatus and BFT. This technique is similar to the modified Larson technique; however, it requires passage through different knee layers and has been classified as anatomic, although a single fibular tunnel is made, making it functionally partially anatomic [96].
- Subsequently, AR was reported by LaPrade in 2010 [97]. The method consisted of drilling two femoral sockets for fixation and two tunnels (one tibial and one fibular) for graft passage and fixation, thus restoring the PLC by reconstructing the FCL, PT, and PFL (Figure 4). Subsequent studies reported the use of different fixation methods [98].

- Kim et al. performed a modified Hughston technique for the anatomical reconstruction of the FCL and PT by drilling one tibial tunnel, one fibular tunnel, and two femoral sockets with different conformations and fixation points (Figure 5) [86,99,100].

Jackson et al. reported higher failure rates of NAT (0–36%) than AR (0–12.1%) through a subgroup analysis [95]. Recently, an all-anatomic open procedure to perform the AR of all three components has been described [101]; however, the literature seems to be rapidly progressing towards all-arthroscopic, all-anatomic procedures [6], which are addressed in the following paragraph.

(3) Arthroscopic reconstructions

Medicine has evolved towards both minimal invasiveness and a reduced surgical time. When considering the anatomical reconstruction of the three main PLC structures, anatomical landmarks should be identified. Thus, it is often not practical when considering open surgery as it means highly invasive procedures. Interestingly, the FCL, PT, and PFL present arthroscopically identifiable landmarks, and their reconstruction is feasible through an all-anatomic, all-arthroscopic approach.

**Figure 6:**
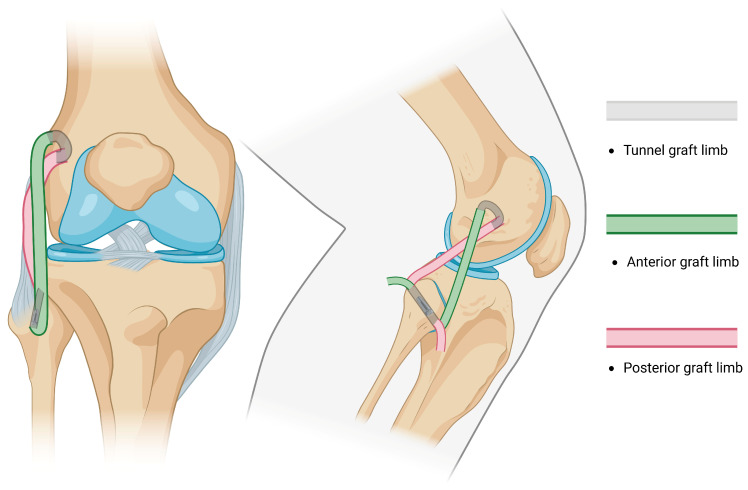
An illustration of the Ahn-Jang arthroscopic reconstruction technique: arthroscopic technique, the femoral portion of the graft is tunneled in a subcortical fashion and the graft ends are fixed at the fibular tunnel.

- The partial arthroscopic PLC approach was described as early as 2009 [102] and considered a popliteus complex reconstruction. Additional open surgery was performed for PCL reconstruction. Several studies later reported the reconstruction of the PFL and PT [103,104].
- Arthroscopic non-anatomical techniques [105] have been used to treat posterolateral rotatory instability in cases of an intact anatomical but non-functional PLC [106]. Arthroscopic NAT involves the stabilization of the posterolateral joint capsule with the lateral meniscus, yet has been reported to influence meniscal excursion.
- In 2019, a partial anatomic technique that reconstructs the FCL and PT only [107], as well as Arciero-based arthroscopic techniques [108], were reported.
- A true all-anatomic, all-arthroscopic technique was first described by Ahn et al., in which the reconstruction of the FCL, PT, and PFL was performed arthroscopically [6], followed by Kolb [109]. The Ahn technique requires the arthroscopic identification of landmarks, expertise in arthroscopic techniques and accessory portals, and extensive knowledge of the posterolateral knee anatomy. Once the landmarks are recognized, the sling reconstruction of the PT is performed, while both ends of the graft (FCL and PFL) are fixed at the fibular tunnel (Figure 6).

The advantage of all-arthroscopic approaches is their minimal invasiveness and early rehabilitation, the clear landmark identification that is not possible through an open approach, and the lower risk of infection. However, not all arthroscopic approaches are anatomic, and what cannot be seen might become more dangerous than what is seen. Despite the fact that the recent literature confirms the surgical safety of arthroscopic PLC reconstructions [60], we believe that, when considering the anatomical PLC as a functional unit, mini-invasiveness can be considered safe when guaranteeing a functional outcome.

Surgical duration seems to be significantly shorter in the arthroscopic Arciero-based technique than in the open Arciero technique [110]; however, this parameter has not been studied for all techniques and is highly dependent on the single case and operator expertise, and should be considered if a different technique is chosen. Between the arthroscopic and open techniques as well as biomechanical validity, arthroscopic PLC anatomic reconstruction has been proven to provide a functional unit and thus restore normal knee biomechanics [111]. Reconstructions aim to stabilize the varus and rotational laxity of the native PLC; a recent meta-analysis performed on biomechanical studies found no difference between fibular-based and tibio-fibular-based reconstructions. Nevertheless, PLC is a multidirectional stabilizer that exerts function in a mobile human knee [112].

The syntheses of the major reconstructions and their advantages, as well as the most recent comparative studies in the literature, are listed in Table 3 and Table 4.

**Table 3:** Summary of advantages and disadvantages of mostly used PLC reconstruction techniques.

Name	Reconstruction	Advantage	Disadvantage
**Albright et al.** [83] **1994**	- Open - PT and FCL - Non-anatomic	- Single tunnel - Does not require extensive expertise - First reconstruction described with reported outcomes in the literature	- Non isometric reconstruction - Non anatomic, with inferior outcomes compared to recent techniques
**Kim et al.** [85] **2001**	- Open - Partial-anatomic - PT and FCL	- Isometric reconstruction	- No pure varus resistance - Open procedure - No true varus stabilization: FCL portion becomes posterior - Requires multiple fixations - Additional anterior non-anatomical structure is present - Peroneal nerve injury risk
**Fanelli-Larson et al.** [90] **2002**	- Open - Partial-anatomic - PT and FCL	- Simple - Single tunnel on fibula	- Forces are more concentrated on the lateral portion as PFL is not reconstructed - Peroneal nerve at risk during fibular tunnel drilling
**Arciero** [96] **2005**	- Open - Partial-anatomic - PT and FCL	- Simple - Single tunnel on fibula - Passage through different layer	- Forces are more concentrated on the lateral portion as PFL is not reconstructed - Peroneal nerve at risk during fibular tunnel drilling
**LaPrade et al.** [97] **2010**	- Open - Anatomic - PT, PFL, and FCL	- Reconstruction with good reported outcomes - Reconstructs all structures offering true posterolateral stability	- Complex - Peroneal nerve at risk during fibular tunnel drilling - Extensive, requires several tunnels and fixations
**Ahn-Jang et al.** [6] **2019**	- PT, PFL, and FCL - Anatomic	- Arthroscopic - Reconstructs all structures offering true posterolateral stability	- Complex, recent - Requires arthroscopic expertise - Time consuming - Peroneal nerve injury risk

PT, popliteus tendon; FCL, fibular collateral ligament; PFL, popliteofibular ligament.

**Table 4:** Synthesis of comparative literature addressing surgical technique, graft choice, and clinical outcomes.

Reference	Technique	FU Duration	Graft	Results
**Yoon et al.** [113] **2011** **Level III**	Semi-Anatomic reconstruction: Tibiofibular technique with PT versus without PT reconstruction	24 months	Achilles tendon allograft	Retrospective study on 32 patients: 17 with PT reconstruction, 15 without PT. Varus stress radiographs significantly improved in both groups. No preoperative or postoperative differences between the groups. Popliteal tendon reconstruction had no effect on anatomic reconstruction stability and clinical results.
**Van Gennip et al.** **2020** [114] **Level IV**	Larson vs. LaPrade (nonanatomic vs. anatomic)	24 months	-	11 Larson reconstructions were compared with a different study with LaPrade reconstruction. PROMs improved significantly. Median varus laxity of the injured knee on varus stress radiographs improved significantly, but did not return to the level of the uninjured knee. In comparison with LaPrade reconstruction, no statistically significant differences in clinical outcome were observed.
**Yeatts et al.** **2021** [115] **Level IV**	Larson vs. LaPrade PLC reconstruction	12+ months	Allograft	Fibular-based technique (350 knee) and tibiofibular-based technique (593 knees). No statistically significant differences in subjective or objective clinical outcome measurements after fibular-based versus combined TF-based PLC reconstruction were observed.
**Sharma et al.** **2021** [116] **Level II**	Modified Larson vs. LaPrade (partial anatomic vs. anatomic)	24 months	Hamstring autografts	Prospective study of 25 patients, 12 LaPrade versus 13 modified Larson reconstructions. Both techniques show good clinical results and restore varus and rotational stability of knee in grade III posterolateral corner injury. No statistical difference between groups.
**Wiess et al.** **2023** [117] **Level II**	Arthroscopic Arciero vs. Arthroscopic LaPrade (partial-anatomic vs. anatomic)	12 months	-	Prospective study of 19 patients. Arthroscopic Arciero patients showed significantly higher maximum flexion angles compared with Arthroscopic LaPrade (134.17° ± 3.76° vs. 126.60° ± 4.22°; *p* = 0.021) at 12 months. Duration of surgery was significantly longer in LaPrade than in Arciero group (121.88 ± 11.63 vs. 165.00 ± 35.65 min; *p* = 0.003). PROMs showed no significant differences between groups. Complications: Arciero group had a dislocation of femoral PCL button while LaPrade presented arthrofibrosis requiring revision.
**Khalis et al.** **2023** [118] **Level IV**	Fibular versus Tibiofibular reconstructions	24+ months	Autologous Gracilis and/or Semitendinosus Achilles/Tibialis posterior allograft	Meta-analysis on 183 patients (90 fibular-based, 93 tibiofibular-based reconstructions). There was no difference between PROMs at 20.3 months. The techniques were equally effective in restoring varus and rotational stability.
**Fahlbusch et al. (2024)** [110] **Level II**	Open Arciero versus Arthroscopic Arciero (open partial anatomic vs. arthroscopic partial anatomic)	14.9 ± 7.2 months	Autologous Gracilis (mentioned in Open Arciero)	Prospective study of 26 patients: 12 Open Arciero versus 14 Arthroscopic Arciero. No clinically relevant differences in PROMs were shown in both groups. Arthroscopic reconstruction showed significantly shorter operation time (*p* = 0.0109).
**Colatruglio et al.** **2024** [119] **Level IV**	Tibial- versus fibular-based PLCR	39.6 months	-	Analysis of tibial- and fibular-based posterolateral corner reconstruction suggests no clinical difference. Four studies reported both tibial- and fibular-based PLCR were found to have no significant differences in PROMs.
**Jackson et al.** **2024** [95] **Level IV**	Anatomic versus non-anatomic techniques	24 months	Hamstrings, Tibialis posterior allograft Biceps tendon autograft	Systematic review of 230 patients; 80% (n = 8/10) of study cohorts performed anatomic reconstruction technique. Failure rates range from 4.3% to 36%. Subgroup analysis revealed a failure rate of 4.3–24.2% for anatomic reconstruction techniques, and 0–36% failure rate for non-anatomic reconstruction. Arthrofibrosis was the most common complication (range, 0–12.1%) following surgery; 0–8% of patients require revision PLC surgery.

PT, popliteus tendon, IKDC, International Knee Documentation Committee, VAS, visual analog scale, PCL, posterior cruciate ligament, PROMs, patient-reported outcome measures.

The table above includes recent clinical trials with reported outcomes of the following reconstruction techniques: open Arciero, open LaPrade, open modified Larson, and arthroscopic Arciero. The systematic review analysis included in the table does not provide information regarding the specific technique utilized, which may have influenced the significance of the relative statistical analysis. Among the remaining studies, Yoon et al.’s [113] study compared patients who underwent anatomic PLC reconstruction including PT reconstruction with those who did not receive PT reconstruction reporting equivalent patient radiographic and clinical outcomes 24 months following surgery. Similarly, Van Gennip et al.’s [114] and Sharma et al.’s [116] studies show no statistical difference between LaPrade and Larson/modified Larson reconstructions after 24 months.

Yeatts et al. [115], Khalis et al. [118], as well as Colatruglio et al. [119] compared fibular- and tibial-based PLC reconstructions at 12 months, 24 months, and 39.6 months, respectively, and all found no statistical difference in patient subjective or objective outcomes between groups.

**Figure 7:**
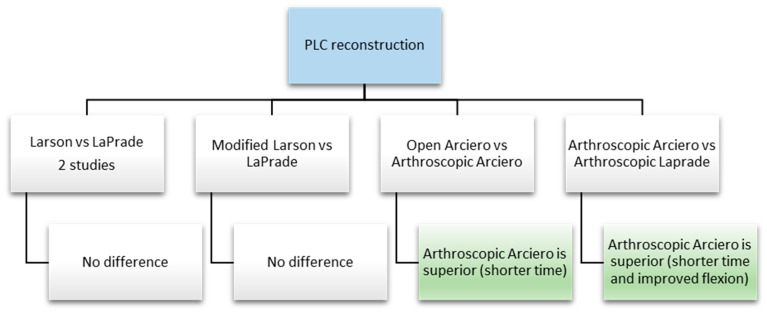
Synthesis of recent comparative papers that specify the surgical technique implied.

Overall, there seems to be no difference between reconstructions, except for three studies that address Larson, LaPrade, and Arciero techniques: the arthroscopic Arciero-based technique led to improved flexion in one study [117] and a shorter surgical time in two studies (Figure 7) [110,117].

The authors thus conclude that if an arthroscopic approach is chosen, it should be anatomical; otherwise, conversion to partial-anatomic open techniques that offer functional outcomes, such as the modified Larson or Arciero techniques, is preferred. The types, advantages, and disadvantages of the grafts used in PLC reconstruction are summarized in Table 5.

The PLC is a complex anatomical entity that is regarded as a functional unit, not merely an anatomical structure. When evaluating patients with possible functional impairment, a multilevel assessment should be conducted, starting with the clinical history and extending to a thorough clinical and instrumental examination, and surgical plan. Efforts should be made to select an adequate graft and to anatomically reconstruct the involved PLC structures whenever feasible. Finally, postoperative rehabilitation and follow-up are crucial to guarantee a good quality of life, early return to sports, and appropriate functional outcomes.

## 8. Postoperative Rehabilitation

Patients undergoing PLC reconstruction require a dedicated rehabilitation program that addresses protection and the timing of weight bearing (WB), assessing the ROM, regaining strength and balance, as well as adequately preparing the athletes to return to sports.

Morris et al. [12] addressed the postoperative management PLC reconstruction with combined ligamentous injuries by comparing immediate WB, progressive WB (partial to full WB by 6 weeks postoperative) and delayed WB (starting 4 weeks postoperative). The time to return to sports did not differ significantly between groups (9 months); however, it tended towards an earlier return in the progressive WB group (6 months). Complications were significantly less in the progressive WB group (3%) compared with delayed WB (44%) and immediate WB (25%) groups.

In the setting of PLC reconstruction, it is necessary to formulate a specific training program based on the associated ligamentous injuries. The early postoperative phase (0–6 weeks) should allow progressive weight bearing, and the knee should be protected with a locked brace at 0° during ambulation and sleeping, while avoiding tibial rotation and varus forces. Exercises in this phase opt for a progressive ROM (Ex: supine/wall slides, strap aid, and patellar mobility) and neuromuscular control (Ex: QM isometrics, prevent QM inhibition, prone straight leg raise/prone knee extension, electrostimulation, seated/supine core, and thigh strengthening). Once symptoms are controlled and a pain-free 0–90 ROM is reached, a supine leg raise is performed without extensor lag, and 75% WB is possible, phase 2 can be safely initiated [77,127,128,129].

Between 7 and 12 weeks, full WB is encouraged and walking aids may be discharged once there is active QM activation and strength, ROM should be further assessed (Ex: high saddle stationary bicycle), and balance training can be initiated. In this phase, closed-chain exercises and core/endurance are reinforced. When compared to the contralateral side, once a normal gait, a 0–130° ROM, and 80% muscle performance (or 8 s ascend/6 s descend steps) are reached, the rehabilitation can proceed towards the 3rd phase [77,130].

Between 13 and 20 weeks, dynamic strength and plyometrics are encouraged (Ex: leg press/squat 80–0°, step machine, multiplanar surfaces, forwards running, and active knee extension of 80–0°). Once core exercises can be performed without lumbo-pelvic or femoral compensations, and a dynamometry of 85–90% is reached, a return to sports can be considered following the assessment of sport-specific demands. When returning to sports (>20 weeks), antigravity training rehabilitation, sport-specific exercises, as well as full-body running are encouraged [77,130,131,132].

We believe that the research on the PLC is ongoing and would greatly benefit from motion analysis techniques that will aid physicians establishing patient-centred care for better clinical and surgical choice, as well as treatment outcomes. Future high-quality comparative trials addressing long-term outcomes of PLC treatments, as well as research focusing on a biomechanical load analysis of the isolated PLC structures are essential to improve the current algorithms and lead to standardized treatment protocols and satisfactory outcomes for patients affected with PLC injury.

## 9. Conclusions

The PLC of the knee joint is a complex anatomical functional unit that includes various ligamentous and tendinous structures that are crucial for joint stability. To achieve better treatment for PLC injuries, a thorough understanding of the anatomy, biomechanics, and function of the PLC is essential. PLC injuries, commonly associated with cruciate ligament injuries, should be quickly diagnosed because failure to do so can lead to ongoing instability, secondary OA, and graft failure of the cruciate ligament. Based on a literature review, no reconstruction technique clearly stands out as superior. As our understanding of the anatomy, biomechanics, and clinical aspects of PLC injuries advances, striking a balance between restoring the native anatomy and safely performing PLC reconstruction remains an important challenge. While there has been an increase in publications on arthroscopic PLC reconstruction, most studies still have low levels of evidence and offer limited insights into optimal treatment options. Anatomical PLC reconstruction is believed to provide better clinical outcomes, and treatment should be tailored to the individual condition of each patient.

## Figures and Tables

**Table 1:** The function and anatomic course of each structure of the PLC.

Structure [References]	Direction of Stabilization	Range of Stabilization	Anatomic Course
**Fibular collateral ligament (FCL)** [18,28,29,30,38]	Varus	Maximal primary stabilizer at 0° gradually decreasing until 90°	Origin 1.4 mm proximal 3.1 mm posterior to the lateral femoral epicondyle Insertion Anteriorly on the fibular head 28.4 mm distal to the fibular styloid tip
	External rotation	Maximal function around 0–30°	
	Tibial translation	Secondary multidirectional stabilizer of tibial translation	
**Popliteus tendon (PT)** [19,33,39] **PT is essential to unlock flexion by internally rotating the tibia and externally rotating the femur**	Varus	Secondary function against varus angulation between 20 and 60° *	Origin around 18.5 mm anteriorly (17–23 mm) from the FCL attachment Insertion Posteromedial proximal tibia
	Rotation	Primary function against external rotation emphasized between 30 and 90° * Secondary minimal function against internal rotation at all angles *	
	Tibial translation	Secondary function against anterior tibial translation between 0 and 30° *	
**Popliteofibular ligament (PFL)** [34,35,37]	Varus	Small but significant effect between 0 and 30° *	Origin 5–10 mm distal to the lateral femoral epicondyle, expanding from the popliteus muscle-tendon Insertion 20–40 mm below the tibial plateau at the posterolateral fibular head
	External rotation	Significant effect between 0 and 90°: favours external rotation in full extension, reduces it between 30 and 90° *	
	Tibial translation	Variable yet significant effect that seems to depend on coupled directional forces at different degrees of knee flexion *	
**Ileotibial band (ITB)** [20,40,41,42] **Knee extensor between 0–30 and flexor above 40°**	Varus	Prominent role against varus angulation during extension, the tract is tightest between 10 and 30°	Origin 20–30 mm from antero-superior iliac crest Insertion 20–40 mm distal to tibial plateau at Gerdy’s tubercle
	Tibial rotation	Produces 2.4 degrees of external rotation when activated *	
	Tibial translation	During ITB activation, there is less anterior tibial translation	
**Biceps femoris tendon (BFT)** [20,25,43,44]	Varus	Secondary role	Origin Long head: 100–120 mm below PSIS, from ischial tuberosity Short head: Lateral tip of the linea aspera, 120–150 mm from femoral head Insertion 10–15 mm below the fibular head
	Tibial rotation	Lateral hamstring activity peaks with external rotation *; activation causes external rotation	
	Tibial translation	Secondary role of resistance against anterior tibial translation at 90° crucial in extension to lower ACL strain	
**Arcuate complex (AC)** [22,23]	Varus stress	Secondary function	Considered as a thickening and merge of structures and not a single ligament on its own Origin Posterolateral femoral condyle, 10–20 mm below FCL Insertion Posterior fibular head
	Rotational stability	Primary function against external tibial rotation	
	Tibial translation	Secondary function reducing posterior tibial translation, reduces strain on PCL	
**Posterolateral capsule (PC)** [24,45]	Varus	Secondary role	Origin Posterolateral femoral condyle and lateral intercondylar notch, connects to LCL and popliteal groove Insertion fibular head 10–15 mm from fibular head tip
	Rotational stability	Static stabilizer against external tibial rotation	
	Posterior tibial translation	Anatomical passive resistance to posterior tibial translation is extension, and increases overall with posterior forces between 30° and 75°	
**Lateral gastrocnemius muscle (LGM)** [42,46]	Varus	Influences 1.28–1.42° of varus and valgus at different degrees of ROM	Origin 50–70 mm superior to joint line from the lateral femoral condyle Insertion 20–30 mm below the calcaneal tuberosity at the posterior calcaneus Achilles insertion
	Rotational stability	Significantly limits rotation with variable effect based on knee flexion degrees; −8.0° at 90–100° flexion and +4.81° at 20–30° flexion	
	Tibial translation	Significantly limits anterior–posterior lateral drawer effect by 18.64 mm at 90–100° in conjunction with ITB	
**Quadriceps muscle (QM)** [36,47,48,49,50] **Synergetic effect, not a part of PLC**	Varus	Resists both varus and valgus stresses through patellar tendon	Origin AIIS, supra-acetabular region of acetabulum (rectus femoris) Greater trochanter, lateral lip of linea aspera (vastus lateralis) Medial linea aspera, medial intermuscular septum (vastus medialis) Anterolateral femoral shaft (vastus intermedius) Insertion Tibial tuberosity as patellar tendon
	Rotational stability	Limits both internal and external rotation through patellar tendon; however, internal rotation is elicited by vastus medialis obliquus	
	Tibial translation	Mostly leads to anterior tibial translation between 0 and 80° and slightly favors posterior tibial translation between 80 and 120°.	

* Statistically significant reported data, AIIS: anterior inferior iliac spine. From the above-reported functions, we may conclude that all components of the functional unit exert multidirectional stabilization to a variable extent and that rotational stability is maintained by more structures than varus or translational stabilities. This tendency may explain the higher rates of true rotational failure following partial or non-anatomical ligamentous reconstructions. The syntheses of the primary stabilizing structures of the functional unit are presented in Table 2.

**Table 2:** Functional unit of the PLC.

Prominently Acting Forces		
Varus Stability	Rotational Stability	Translational Stability
**FCL** **ITB**	FCL PT PFL BFT AC LGM	QM PC

FCL, fibular collateral ligament; ITB, iliotibial band; PT, popliteus tendon; PFL, popliteofibular ligament; BFT, biceps femoris tendon; AC, arcuate complex; LGM, lateral gastrocnemius muscle; QM, quadriceps muscle; PC, posterolateral knee capsule.

**Table 5:** Summary of the advantages and disadvantages according to graft type.

Graft Type	Advantage	Disadvantage
**Autograft** - **Gracilis/Semitendinosus** [120] - **Long Head BFT**	Economic No host reaction Reduced risk of infection Biologically advantageous No risk of disease transmission BFT can be used for multiple ligamentous reconstructions Semitendinosus strength 1060 N Gracilis strength 838 N	Limited availability Usually available for a single reconstruction Longer surgical time Additional scar Donor site morbidity Fewer reported trials compared with allografts BFT is part of the PLC; its use may weaken the PLC in certain degrees of ROM A hip flexor is implicated which means possible movement limitation and reduced return to sports owing to altered quadriceps/hamstring strength Alteration of the long-head BFT predisposed to injury and delayed return to sports [121]
**Allograft** - **Achilles tendon** [122] - **Tibialis anterior/Tibialis posterior** [123] - **Semitendinosus** [124]	Can be used for multiple reconstructions Availability No donor site morbidity	Economic burden Risk of host reaction Higher risk of infection Not available in all countries Limited biological integration
**Synthetic graft** - **LARS sports ligament** [125] - **FiberTag TightRope** [126]	Can be used for multiple reconstructions Availability No donor site morbidity	Economic burden Rare reaction of host Non-biological, thus bears a high risk of subsequent infection Rejection response/biocompatibility Tissue integration is limited Biomechanical performance

BFT, biceps femoris tendon; PLC, posterolateral corner; ROM, range of motion; N, newton.

## Data Availability

Data available on request from the authors.
